# Characterization of the complete chloroplast genome of *Aquilaria sinensis*, an endangered agarwood-producing tree

**DOI:** 10.1080/23802359.2019.1703593

**Published:** 2020-01-08

**Authors:** Xin Deng, Zhenxing Jiang, Qingbin Jiang, Wei Guo, Yongquan Li, Xianzhi Zhang

**Affiliations:** a College of Horticulture and Landscape Architecture, Zhongkai University of Agriculture and Engineering, Guangzhou, China;; b Research Institute of Tropical Forestry, Chinese Academy of Forestry, Guangzhou, China

**Keywords:** *Aquilaria sinensis*, agarwood, conservation genomics, phylogenomics

## Abstract

*Aquilaria sinensis* is one of the most important agarwood-producing trees but critically endangered at present. In this study, we produced the complete chloroplast (cp) genome of *A. sinensis* via genome survey analysis. The assembled genome is 174,914 base-pairs (bp) in length, with one large single-copy region of 87,361 bp and one small single-copy region of 3347 bp separated by two inverted repeats of 42,103 bp. The genome contains a total of 142 genes, including 96 protein-coding genes, 8 rRNAs, and 38 tRNAs. The phylogenomic tree strongly supports *Aquilaria* as a monophyly and *A. sinensis* sister to *A. yunnanensis*.

Agarwood is widely used as traditional drug, interior decoration and valuable perfume for thousands of years in Asia (Hashim et al. 2016). The genus *Aquilaria* is well known as the main material of the agarwood (Lee et al. 2016). Unfortunately, due to the overharvesting of wild resources for high demand of agarwood products, all *Aquilaria* species are currently endangered and regulated under the CITES (http://checklist.cites.org). Especially, the species *Aquilaria sinensis* (Lour.) Gilg is critically threatened and has been listed as ‘Vulnerable (VU)’ by the IUCN Red List (http://www.iucnredlist.org). Despite its great value, the genomic resources for *A. sinensis* are rather limited, which constraints the conservation actions for this species (Farah et al. 2018). In this study, we produced the complete chloroplast (cp) genome of *A. sinensis*, to facilitate further conservation genomic studies in agarwood-producing tree species.

Fresh leaves of *A. sinensis* were collected from Arboretum of Zhongkai University of Agriculture and Engineering in Guangzhou, Guangdong province of China (113°16′35″E, 23°06′35″N), to extract genomic DNA. The voucher specimen was deposited at the Tree Herbarium of Zhongkai University of Agriculture and Engineering (accession number ZXZ19010). A paired-end (PE) library was constructed and sequenced using Illumina platform (HiSeq 2500) at Novogene in Beijing, China. The cp genome was assembled by CLC Genomics Workbench v7.5 (CLC Bio, Aarhus, Denmark), as stated previously (Wang et al. 2018). Subsequently, we annotated the assembled cp genome via DOGMA (Wyman et al. 2004).

The finished cp genome of *A. sinensis* (GenBank accession MN720647) is 174,914 base-pairs (bp) in length, showing a typical quadripartite organization: one large single-copy region (LSC) of 87,361 bp and one small single-copy region (SSC) of 3,347 bp separated by two inverted repeats (IRs) of 42,103 bp. The cp genome contains a total of 142 genes, including 96 protein-coding genes, 8 ribosomal RNA (rRNA) genes, and 38 transfer RNA (tRNA) genes. There are 10 protein-coding genes that contain introns (*atpF*, *ndhA*, *ndhB*, *petB*, *petD*, *rpl2*, *rpoC1*, *rps12*, *rps16*, *ycf3*). The overall GC content of this cp genome is 36.7%. Interestingly, we found that the *A. sinensis* cp genome generated here has a relatively short SSC region (3,347 bp) and two long IR regions (42,103 bp), inconsistent with the result of Wang et al. (2016). The divergent cp genome structure suggested that cp genome may vary largely in *A. sinensis* populations.

Phylogenetic position of *A. sinensis* was further estimated by RAxML v.8.2.8 (Stamatakis 2014) using 12 cp genomes of Thymelaeaceae and relatives. The resulted maximum likelihood (ML) tree supported that all the species of the genus *Aquilaria* formed a monophyletic clade with 100% support (Figure 1). The relationships with *Aquilaria* were clearly resolved as ((((*A. sinensis*, *A. yunnanensis*), *A. malaccensis*), *A. crassna*). The clade of two *A. sinensis* cp genomes was also highly supported (Figure 1), confirming the validity of the obtained cp genome of *A. sinensis* in this study. In all, the cp genome phylogenomics largely resolved the phylogeny of Thymelaeaceae, consistent with previous studies (Lee et al. 2018; Li et al. 2019).

**Figure 1.**
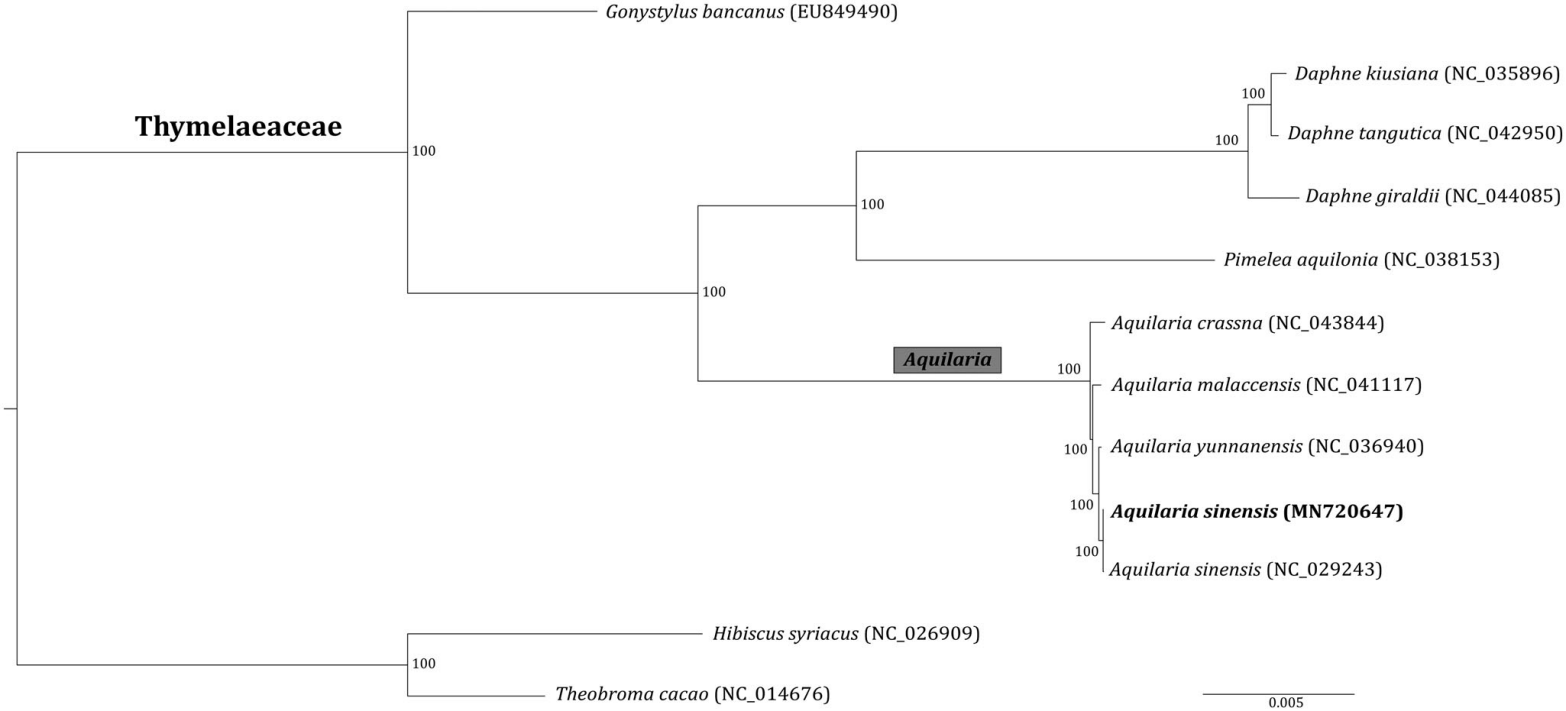
Maximum likelihood tree inferred from 12 chloroplast genomes. The position of *Aquilaria sinensis* sequenced in this study is shown in bold. Numbers at nodes are bootstrapping support values.
